# Vitamin D Supplementation in Exclusively Breastfed Infants Is Associated with Alterations in the Fecal Microbiome

**DOI:** 10.3390/nu14010202

**Published:** 2022-01-01

**Authors:** Tengfei Ma, Sihan Bu, Nigel Paneth, Jean M. Kerver, Sarah S. Comstock

**Affiliations:** 1 Department of Epidemiology and Biostatistics, College of Human Medicine, Michigan State University, East Lansing, MI 48824, USA; matengfe@msu.edu (T.M.); kerverje@msu.edu (J.M.K.); paneth@msu.edu (N.P.); 2 Department of Food Science and Human Nutrition, Michigan State University, East Lansing, MI 48824, USA; busihan@msu.edu; 3 Department of Pediatrics and Human Development, College of Human Medicine, Michigan State University, East Lansing, MI 48824, USA

**Keywords:** infant gut microbiota, feeding practices, diet, breastfeeding, vitamin D supplementation, cohort

## Abstract

Breastfeeding and introduction of solid food are the two major components of infant feeding practices that influence gut microbiota composition in early infancy. However, it is unclear whether additional factors influence the microbiota of infants either exclusively breastfed or not breastfed. We obtained 194 fecal samples from infants at 3–9 months of age, extracted DNA, and sequenced the V4 region of the 16S rRNA gene. Feeding practices and clinical information were collected by questionnaire and abstraction of birth certificates. The gut microbiota of infants who were exclusively breastfed displayed significantly lower Shannon diversity (*p*-adjust < 0.001) and different gut microbiota composition compared to infants who were not breastfed (*p*-value = 0.001). Among the exclusively breastfed infants, recipients of vitamin D supplements displayed significantly lower Shannon diversity (*p*-adjust = 0.007), and different gut microbiota composition structure than non-supplemented, breastfed infants (*p*-value = 0.02). MaAslin analysis identified microbial taxa that associated with breastfeeding and vitamin D supplementation. Breastfeeding and infant vitamin D supplement intake play an important role in shaping infant gut microbiota.

## 1. Introduction

The gut microbiota has been considered an “invisible organ” of the human body, playing important roles in modulating host functions, including metabolism, digestion, and gut mucosal immune responses and integrity [1,2]. Dysbiosis of the gut microbiota may be associated with various adverse health outcomes in infants such as asthma, Crohn’s disease, inflammatory bowel disease, and type 1 diabetes (T1D) [3,4,5,6,7]. The colonization of gut bacteria begins at birth and remains remarkably dynamic until about 2–3 years of age when more stable microbial profiles begin to emerge [8,9]. In addition to the mode of delivery and antibiotic exposure, infant feeding practices are key factors in shaping early microbiota composition [10,11]. Recent studies have shown that gut microbial profiles in breastfed infants are significantly different from those in formula-fed infants and change rapidly after the transition from breastfeeding to formula or solid food [12,13,14,15]. The differences in gut microbiota composition observed between formula-fed and breastfed infants have, at least partially, been attributed to the absence of human milk oligosaccharides (HMOs) in infant formula [16]. Human milk is enriched with HMOs, which have been linked to beneficial bacteria in the gut microbiota [17,18]. The introduction of solid food represents another key factor influencing the composition of infant gut microbiota, producing an adult-type complex microbiome dominated by the phyla Bacteroidetes and Firmicutes [8,19].

Most gut microbiota research on infant nutrition to date has focused on breastfeeding and the introduction of solid food. Little is known about the effect of other dietary features within the two different feeding practices. It is recommended that infants who are breastfed exclusively should take vitamin D supplements every day due to the variability of vitamin D content in human breastmilk [20]. As all infant formula in the United States is fortified with vitamin D, infants who are fed exclusively with formula usually do not need vitamin D supplementation. Vitamin D not only prevents rickets, but also plays an important role in immune responses and metabolic processes that maintain the integrity of the gut epithelium [21,22,23]. 

Human milk can also be provided by bottle, from banking of milk by the mother or from human milk banks. This indirect form of breastfeeding can lead to enrichment by environmental bacteria, such as *Stenotrophomonas* and *Pseudomonadacea* [16]. The water used to reconstitute powdered infant formulas may also be an important exposure for infant health outcomes. Reconstituting formula with tap water can lead to excessive fluoride and lead intake [24,25]. Different water types (e.g., city water, well water, filtration systems) can be a source of varied bacterial composition. 

We sought to determine the association between maternal and infant characteristics and infant feeding practices and the gut microbiota profiles in 3- to 9-month-old infants. In addition, we analyzed the exclusively breastfed and non-breastfed infants separately to assess the impact of unique feeding practices features on the microbiotas of the infants in each group. 

## 2. Materials and Methods

### 2.1. Study Participants

The study population was drawn from the Michigan Archive for Research on Child Health (MARCH) cohort [26], an ongoing population-based pregnancy and birth cohort set in Michigan’s lower peninsula. The purpose of the MARCH study is to store biological specimens and other health information in pregnancy and early life that can be used to better understand the causes of problems in pregnancy and optimize the health of children. This cohort is a component of a nation-wide study of child health called the Environmental influences on Child Health Outcomes (ECHO) [27]. Our analysis included mothers who provided informed consent for providing infant stool samples. During the MARCH 3 month phone interview mothers confirmed their interest in participating in this sample collection. The fecal Collection kits were assembled at the lab and sent to the participants via mail. The collection kits included instructions for taking a fecal sample, an OMNIgene-GUT tube for sample collection, a box with postage to return the sample, and diapers for the infant sample. Samples were returned to the lab by mail, and fecal aliquots were stored at −80 °C upon reaching the lab. 194 fecal samples have so far been collected from singleton infants aged from 3 to 9 months old. The infants in this analysis were 3–9 months of age between 2018 and 2021.

### 2.2. Data Collection

Several questionnaires were administered to mothers from the first prenatal visit through 9 months postpartum. The questionnaire at the first prenatal visit included demographic information about the mothers, their breastfeeding plans and many health-related practices and conditions as well as their estimated due date. Infant dietary feeding patterns, including breast milk and/or formula, detailed feeding practices, and complementary food intake, were collected at the same time as the fecal samples. Detailed information, including the infant’s sex, birth weight, complications of pregnancy, mode of delivery (vaginal vs. C-section), pre-pregnancy BMI and gestational age, was abstracted from the birth certificate.

### 2.3. Fecal Microbiota Analysis

Once received in the lab, the fecal samples were aliquoted into sterile tubes and stored at −80 °C. DNA was extracted following a modified version of the Human Microbiome Project’s protocol as described previously [28]. Barcoded primers were used to amplify the V4 region of the 16S rRNA gene following the mothur wet lab documentation. PCR amplification also followed the wet lab protocol outlined in the mothur documentation. The resulting 16S rRNA libraries were sequenced using 250 base pair Illumina MiSeq with V2 chemistry at the Michigan State University genomics core. After trimming, clean sequences were analyzed using the QIIME2 (2021. 2 version) pipeline [29]. Demultiplexed sequences were further quality filtered and clustered using QIIME2’s DADA2 plugin to generate the ASV table [30]. A phylogenetic tree was constructed from the sequences using the QIIME2 FastTree plugin with default parameters [31]. Unique amplicon sequence variants (ASVs) were assigned a taxonomy by the QIIME2 feature-classifier plugin, using the Silva 132 database at the similarity threshold of 99% (for 16S data) [32,33]. Samples were rarefied to 6000 sequencing reads per sample, leaving 191 stool samples with 6905 unique ASVs, and findings were summarized at the genus taxonomic level. 

### 2.4. Statistical Analysis

We used multivariate ordinal logistic regression models to estimate the association between pre-pregnancy BMI and breastfeeding practices, with adjustment for demographic variables and delivery mode. 

Gut microbiota is analyzed by alpha diversity (Chao1 index and Shannon index) and beta diversity (Bray–Curtis dissimilarity and Weighted UniFrac) using the “vegan” package in R [34]. The difference of alpha diversity and relative abundance of taxa between feeding practices groups were tested by Wilcoxon rank test and Kruskal–Wallis test with false discovery rate (FDR) correction for multiple comparisons. We assessed the influence of factors significantly associated with gut bacterial community structure by multivariate models using a Permutational Multivariate Analysis of Variance (PERMANOVA) with 999 permutations based on Bray–Curtis dissimilarities (adonis, R vegan package) [34,35]. PERMANOVA is non-parametric multivariate statistical test, with *p*-values obtained using appropriate distribution-free permutation techniques. We used the multivariate association with linear models (MaAsLin) to identify associated microbiological taxa with the feeding practices and other related factors [36,37]. MaAsLin is a multivariate statistical framework that identifies associations between clinical metadata and microbial community abundance and provides both nominal *p*-values and FDR adjusted *p*-values (q-values) by Benjamini–Hochberg procedure [38]. Associations are considered significant when the q-value is below the threshold of 0.1.

## 3. Results

### 3.1. Participants and Feeding Practices

We analyzed gut microbiome samples from 191 infants. In Table 1, maternal and infant characteristics are compared by breastfeeding status (exclusive breastfeeding, partial breastfeeding, and not breastfeeding). During the week immediately preceding stool sample collection, 88 (46.1%) infants were fed exclusively with breast milk, 43 (22.5%) were fed partially with breast milk, and 60 (31.4%) were not fed with breast milk. The median age at the time of specimen collection was 3.8 months (range: 3.0 months–9.3 months). Partially breastfed infants were more likely to be fed with complementary foods than those who were not breastfed (44.2% vs. 35.0%, *p* > 0.4). Infants who were exclusively breastfed were more likely to be given vitamin D supplementation than partially breastfed or not-breastfed infants (39.8% vs. 18.6% vs. 1.7%, *p* < 0.01). A higher proportion of mothers who practiced exclusive breastfeeding were of normal BMI (18.5–25.0) prior to pregnancy comparing to those who practiced partial breastfeeding or who were not breastfeeding (50.0% vs. 39.5% vs. 26.7%, *p* < 0.01). Mothers who practiced exclusive breastfeeding were more likely to have a college degree than women who partially breastfed or did not breastfeed (72.4% vs. 61.9% vs. 32.2%, *p* < 0.01). In a multivariate model adjusted for maternal age, maternal educational level, pre-pregnancy BMI (continuous), delivery mode, and infant age, mothers with higher pre-pregnancy BMI were less likely to practice breastfeeding (OR = 0.95, CI: 0.91–0.99, *p*-value = 0.01, Table 2), and mothers with higher educational level were more likely to practice breastfeeding (OR = 2.66, CI: 1.72–4.21, *p*-value < 0.001, Table 2).

### 3.2. Gut Microbiota Analysis

**Figure 1:**
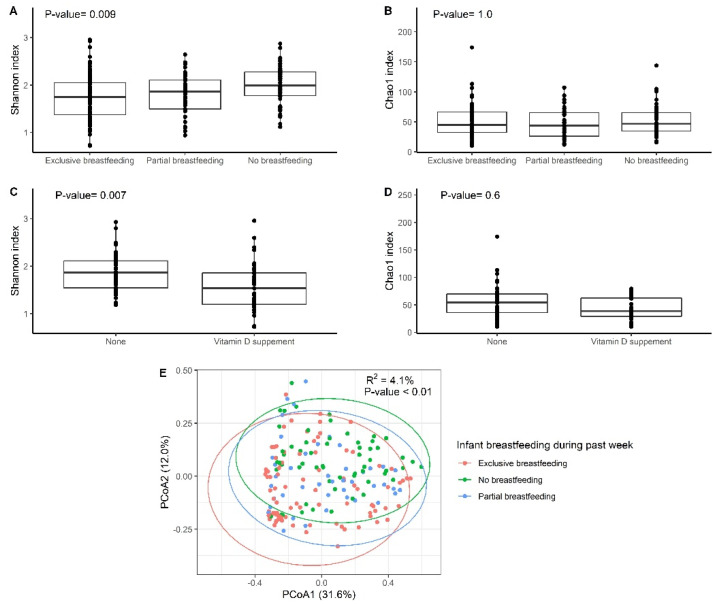
Infant alpha and beta diversity by infant breastfeeding and Vitamin D supplement. FDR adjusted *p*-value for alpha diversity was displayed in upper-left. (**A**) The Shannon index was used for alpha diversity. All the participants were included in the analysis (N = 191). Group differences were tested by Kruskal-Wallis test. We then performed post hoc test for multiple comparisons. After FDR adjustment, no breastfeeding group has significant difference with exclusive breastfeeding (adjusted *p*-value < 0.01). Partial breastfeeding group has no significant difference with exclusive breastfeeding group (*p*-value = 0.4, adjusted *p*-value = 0.9) and no breastfeeding group (*p*-value = 0.03, adjusted *p*-value = 0.09). (**B**) The Chao1 index was used for alpha diversity. All the participants were included in the analysis (N = 191). Post hoc test did not find any significant difference between groups. (**C**) The Shannon index was used for alpha diversity. Only breastfeeding participants were included in this subgroup (N = 92). Group differences were tested by Wilcoxon rank test. (**D**) The Chao1 index was used for alpha diversity. Only breastfeeding participants were included in this subgroup analysis (N = 92). (**E**) Principal component analysis (PCoA) ordinations of variation based on the Bray–Curtis distance matrix for all infants. R^2^ and *p*-value were calculated by univariate PERMANOVA test with 999 permutations.

Fecal samples from infants who were exclusively breastfed displayed lower Shannon diversity than samples from those who were not breastfed (FDR adjusted *p*-value < 0.01, Figure 1A). Samples from infants who were partially breastfed displayed Shannon diversity intermediate between the other two groups, but not significantly different from either (FDR adjusted *p*-value = 0.9). Chao1 index was not significantly different across the three groups (FDR adjusted *p*-value = 1.0, Figure 1B). Among the exclusively breastfed, infants who had been given a vitamin D supplement during the previous 24 h displayed lower Shannon index (FDR adjusted *p*-value < 0.01, Figure 1C) and lower Chao1 index (FDR adjusted *p*-value = 0.6, Figure 1D) than those infants who were not given a vitamin D supplement. 

Whether breast milk was fed directly or was pumped and fed to the infant using a bottle, the Shannon and Chao1 indices of the infant gut microbiota alpha diversity were similar (FDR adjusted *p*-value =1.0 and 0.88, respectively, Figure S1). Among the non-breastfed infants, neither the water type used to reconstitute the formula nor the consumption of complementary food during past 24 h was associated with gut microbiota alpha diversity as measured by the Shannon or Chao1 indices (Figure S1).

When classified by breastfeeding status, the gut microbiota communities of the infants were well separated in principal coordinate analysis (PCoA) based on the Bray–Curtis distance matrix and the observation was statistically significant as assessed by PERMANOVA (R^2^ = 4.1%, *p*-value <0.01, Figure 1E). In addition to the feeding practices, gestational age (R^2^ =4.0%, *p*-value = 0.001) and delivery mode (R^2^ = 2.0%, *p*-value = 0.003) were significantly associated with overall gut microbiome composition (Table 3; multivariate PERMANOVA model on Bray–Curtis distances). The PERMANOVA results were consistent with results from the Weighted UniFrac distance metric (Table S1). We then repeated the PERMANOVA analysis on Bray–Curtis distances within exclusively breastfed and not breastfed infants separately. These subgroup analyses also included additional variables. Accordingly, delivery mode (R^2^ = 3.5%, *p* = 0.01) and infant vitamin D supplement in the past 24 h (R^2^ = 3.4%, *P* = 0.02) were significantly associated with gut microbiota composition in exclusively breastfed infants (Table S2). Among the not breastfed infants, only maternal education level (R^2^ = 4.1%, *p*-value = 0.02) was significantly associated with gut microbiota composition (Table S3). The PERMANOVA results of these two subgroup analyses were consistent with results from the Weighted UniFrac distance metrics (results not shown). 

We further assessed the association between breastfeeding status and 8 most abundant genera by univariate analysis (Figure S2). These 8 most abundant genera were *Bacteroides*, *Bifidobacterium*, *Veillonella, Escherichia-Shigella*, *Ruminococcus gnavus*, *Clostridium sensu stricto 1, Prevotella*, and *Lachnoclostridium*. Exclusive breastfeeding was significantly associated with a higher relative abundance of *Bifidobacterium* (FDR adjusted *p*-value = 5 × 10^−5^) and a lower relative abundance of *Lachnoclostridium* (FDR adjusted *p*-value = 5.6 × 10^−7^). 

MaAsLin results revealed that exclusive breastfeeding was significantly associated with the relative abundance of a set of genera, including *Intestinibacter, Flavonifractor*, *Lachnoclostridium*, *Clostridium innocuum group*, *Lactobacillus*, *Bifidobacterium, etc.* (Table 4). Infant age at sample collection and maternal pre-pregnancy BMI were associated with higher relative abundance of *Lachnospira* and *Alistipes*, respectively (Table 4). Among exclusively breastfed infants, infants who had taken a vitamin D supplement in the previous 24 h had a lower relative abundance of *Haemophilus* (Table 5). Among the not breastfed infants, having taken a probiotic supplement in the past 24 h was associated with higher relative abundance of uncultured *Lachnospiraceae* and *Faecalitalea* (Table 5). Maternal pre-pregnancy BMI was associated with a higher relative abundance of *Alistipes (*Table 5).

## 4. Discussion

Our study was conducted in a population with somewhat higher than average rates of exclusive breastfeeding, with nearly half (46.1%) of the infants exclusively breastfed at the median age of 3.8 months. This percentage is higher than in the Infant Feeding Practices Study II in the US (34% at 3 months) in 2007 [39], while it is close to the percentage in CDC National Immunization Survey in 2018 (46.3% at 3 month) [40]. Although the American Academy of Pediatrics recommends vitamin D supplementation for all breast-fed infants [41], only 39.8% of the mothers in our study followed this recommendation, a lower frequency than that has been found in Canadian and European cohorts in 2009 and 2014, respectively [42,43]. 

We found that higher maternal education level and lower pre-pregnancy BMI were independently and significantly associated with an increased odds of being exclusively breastfed, consistent with previous studies in developed and developing countries [39,44,45]. Breastfeeding initiation and duration are also negatively correlated with high pre-pregnancy BMI [46,47]. These associations may be attributed to the physiological factors such as delayed onset of lactogenesis II and imbalances of hormones [48]. Previous studies have showed that maternal obesity can cause the delayed onset of lactogenesis II (DOL) that is associated with mother’s confidence that her milk is sufficient for her child [49,50]. As a result, it can lead to lower rates of breastfeeding initiation and early termination of exclusive breastfeeding. The associations between maternal BMI and lactation success have been recently reviewed [51,52].

Our study showed the importance of both breastfeeding and infant vitamin D supplements in shaping infant gut microbiota composition. Breastfeeding is significantly associated with both alpha and beta diversity of infant gut microbiota. We observed that the Shannon diversity of partially breastfed infants was between that of exclusively breastfed infants and not breastfed infants, though somewhat closer to the exclusively breast fed, suggesting a dose–response relationship between breastfeeding and Shannon diversity of infant gut microbiota. These results agree with a previous study, which reported that the composition of gut microbiota from partially breastfed infants are similar to that from exclusively breastfed infants [13]. Among the subgroup analysis of exclusively breastfed infants, the alpha and beta diversity results demonstrated that vitamin D supplementation is associated with infant gut microbiota composition. These results are in good agreement with the study of Lei et al. who investigated the role of vitamin D supplement on gut microbiome from 31 exclusively breastfed infants at 4-months-old [53]. Lei et al. showed that vitamin D supplementation is associated with both alpha diversity and beta diversity of infant gut microbiome [53]. Animal studies demonstrate that vitamin D plays a critical role in maintaining the integrity of the intestinal mucosal barrier by preserving the integrity of junctions that control mucosal permeability and reduction of pro-inflammatory cytokines such as IL-8 [23,54,55]. In addition, studies also found that VDR-mediated signaling inhibits inflammation-induced apoptosis of intestinal epithelial cells [54,56]. As a result of these effects on the intestinal mucosa, vitamin D acts as an important factor influencing the gut microbiota. 

Besides the infant feeding practices, the results herein confirm that gestational age, delivery mode and maternal educational level are also significantly associated with gut microbiome composition. These results are consistent with many previous studies [10,13,16]. However, delivery mode is only associated with gut microbiota composition among the exclusively breastfed infants, while no significant association was found in the not breastfed infants. This might be attributed to the fact that C-section delivery can delay lactation initiation [57] and shape the bacterial composition of breast milk [58,59]. Maternal educational level is the only factor that significantly associated with infant gut microbiota composition among the not breastfed infants, whereas it was not significant among the exclusively breastfed infants. This finding suggests that not breastfed infants are more susceptible to socio-economic factors, such as educational level, which is normally connected to offspring diet and nutritional status [60]. Hence, this association among the not breastfed infants might be mediated by the types of solid foods introduced and brands of formula purchased. However, our data set did not allow us to test these associations.

Our study confirmed that *Bifidobacterium* was enriched in breastfed infants when compared with non-breastfed infants. Lower abundance of *Bifidobacterium* in infants due to early cessation of breastfeeding could potentially inhibit the interaction of bifidobacterial-mediated metabolites with the immune system, leading to higher levels of inflammation [61,62]. In contrast, the genus *Lachnoclostridium* (*Lachnospiraceae* family) was found to be enriched in the non-breastfed infants when compared with exclusively breastfed or partially breastfed infants. In addition, the genera *Eisenbergiella* and *Lachnospiraceae_*uncultured, which also belong to *Lachnospiraceae* family were found to be enriched in the non-breastfed infants by MaAslin. These observations agree with previous studies that lower abundance of *Lachnospiraceae* is associated with breastfeeding at 3 months of age [63]. The evidence from many studies showed that *Lachnospiraceae* family or specific genera of *Lachnospiraceae* may be associated with several inflammatory conditions, such as metabolic syndrome, obesity, diabetes, and liver diseases [64,65,66,67]. Thus, our results suggest several possible mechanisms that might explain the beneficial effects of breastfeeding on health outcomes.

Notably, the genus *Haemophilus* was enriched in the breastfed infants. However, exclusively breastfed infants who had taken a vitamin D supplement in the past 24 h had a lower relative abundance of *Haemophilus* compared to those exclusively breastfed infants who were not supplemented. Consistent with our study, Fehr et al. showed that breastmilk may specifically provide *Haemophilus* to the infant gut [13]. Luthold et al. also demonstrated that *Haemophilus* was less abundant in the group of highest vitamin D intake [68], supporting the hypothesis that a reduced immune response in vitamin D deficiency could augment the competitive advantage of *Haemophilus* and influence the composition of the infant gut microbiome [69].

Our study did not detect any effect of feeding with expressed milk, infant antibiotic intake, and water type for formula on gut microbiome. However, sample sizes were small for many of these comparisons. Therefore, pooling data from multiple cohort studies or analysis in larger cohorts with similar data are necessary to confirm this lack of association. For instance, previous studies demonstrated that infants who had been exposed to antibiotics had decreased abundance of *Bifidobacteria* and *Bacteroides* in the infant gut microbiome [70]. In our study, we asked the mothers if the infant had taken any antibiotics since birth, whereas the timing of antibiotics administration was unknown. Hence, it’s possible that infant gut microbiome had recovered from the dysbiosis states caused by antibiotics at the time of stool sample collection. The inconsistent results may also be attributed to variations in the antibiotic type, dosage, duration [71]. 

An important limitation of this study is that only a single stool sample was available for analysis. Although the infant feeding practice information was collected at the same time as the stool sample collection and can demonstrate the impact of short-term exposures on the infant gut microbiota composition, we are unable to determine how these factors contribute to the temporal development of the infant gut microbiome. Another limitation is that we did not collect more detailed information, such as dose of vitamin D, maternal vitamin D status, and timing of antibiotic administration. Future studies would benefit from a longitudinal stool sample collection during infancy and a more detailed infant feeding practices questionnaire that not only collects proximal but also long-term data about infant nutritional exposures. 

## Figures and Tables

**Table 1:** Characteristics of the mothers and infants by breastfeeding status ^1^.

Characteristic	Exclusive Breastfeeding (N = 88)	Partial Breastfeeding (N = 43)	No Breastfeeding (N = 60)	*p*-Value
Infant age at sample collection (day), mean (SD)	115.5 (17.3)	135.9 (39.8)	126.3 (31.9)	< 0.01
Infant had any antibiotics since birth, n (%)	14 (15.9)	5 (11.6)	8 (13.3)	0.40
Consumption of complementary food during past 24 h, n (%)	0 (0.0)	19 (44.2)	21 (35.0)	< 0.01^3^
Infant probiotic supplement ^2^ during past 24 h, n (%)	4 (4.5)	1 (2.4)	3 (3.3)	0.90
Infant Vitamin D supplement during past 24 h, n (%)	35 (39.8)	8 (18.6)	1 (1.7)	< 0.01
Delivery mode, n (%)				
Vaginal delivery	66 (75)	30 (69.8)	36 (60)	0.10
C-section	22 (25)	13 (30.2)	24 (40)	
Infant weight at delivery (gram), mean (SD)	3461 (551)	3336 (529)	3269 (598)	0.10
Infant sex, n (%)				
Male	43 (48.9)	22 (51.2)	30 (50)	0.98
Female	45 (51.1)	21 (48.8)	30 (50)	
Maternal pre-pregnancy BMI, n (%)				
< 18.5	1 (1.1)	0 (0.0)	3 (5.0)	< 0.01
18.5–25	44 (50)	17 (39.5)	16 (26.7)	
> 25–30	24 (27.3)	11 (25.6)	12 (20.0)	
> 30	19 (21.6)	15 (34.9)	29 (48.3)	
Maternal education level, n (%)				
Did not finish high school	0 (0.0)	0 (0.0)	6 (10.2)	< 0.01
High school graduate or GED	4 (4.6)	4 (9.5)	21 (35.6)	
Some college	20 (23.0)	12 (28.6)	13 (22.0)	
College graduate or more	63 (72.4)	26 (61.9)	19 (32.2)	

^1^ Breastfeeding status information was collected at the time of fecal sample collection. Values are mean (SD) for continuous variables or n (%) for categorical variables. Difference by breastfeeding status was calculated using an ANOVA or chi-squared test. ^2^ Including probiotic supplement, kefir and kimchi. ^3^ Post hoc analysis with Bonferroni adjustment showed significant difference in consumption of complementary food between partial breastfeeding and no breastfeeding groups.

**Table 2:** Association between breastfeeding (exclusive, partial, and no breastfeeding) and perinatal characteristics ^1^.

Maternal Characteristics	Proportional Odds Ratio	95% CI	*p*-Value
Maternal age(year)	1.02	0.96–1.09	0.48
Maternal educational level	2.66	1.72–4.21	< 0.001
Pre-pregnancy BMI (continuous)	0.95	0.91–0.99	0.01
Delivery mode (vaginal vs. C-section)	0.56	0.29–1.05	0.07
Infant age (day)	0.99	0.98–1.0	0.07

^1^ A multivariate ordinal logistic regression analysis was performed to assess the association. Variables in the model include maternal age, maternal education level, pre-pregnancy BMI, delivery mode, and infant age.

**Table 3:** Results of Permutational Multivariate Analysis of Variance (PERMANOVA) ^1^ with 999 permutations for all infants.

Variable	F Value	R^2^	*p*-Value
Breastfeeding during past week	4.0	4.10%	0.001 *
Gestational age	2.3	1.20%	0.03 *
Infant sex	1.1	0.50%	0.36
Delivery mode (vaginal vs. C-section)	3.6	1.80%	0.004 *
infant weight at delivery	0.7	0.30%	0.72
Infant probiotic supplement during past 24 h	1.0	0.50%	0.38
Infant had any antibiotics since birth	0.9	1.00%	0.47
Maternal educational level	2.7	1.30%	0.02 *
Maternal pre-pregnancy BMI (continuous)	0.37	0.20%	0.96

^1^ Bray–Curtis distance was used for the PERMANOVA. * indicates the *p*-value < 0.05.

**Table 4:** MaAsLin Analysis Results: Associations of infant feeding practices and gut microbiome taxa at genus level adjusted by covariates in all infants (N = 191) ^1^.

Taxonomy at Genus Level	Meta Data Value	Coefficient	N/N Not 0	*p*-Value	q-Value ^2^
*Intestinibacter*	Exclusive breastfeeding	−0.567	191/64	3.6 × 10^−10^	3.0 × 10^−7^
*Flavonifractor*	Exclusive breastfeeding	−0.896	191/145	4.0 × 10^−8^	1.7 × 10^−5^
*Lachnoclostridium*	Exclusive breastfeeding	−0.998	191/146	1.9 × 10^−7^	5.2 × 10^−5^
*Clostridium innocuum* group	Exclusive breastfeeding	−0.393	191/44	4.9 × 10^−6^	6.8 × 10^−4^
*Lactobacillus*	Exclusive breastfeeding	0.680	191/115	4.3 × 10^−6^	6.8 × 10^−4^
*Lactococcus*	Exclusive breastfeeding	−0.287	191/29	1.7 × 10^−5^	1.7 × 10^−3^
*Bifidobacterium*	Exclusive breastfeeding	0.535	191/186	2.4 × 10^−4^	0.018
*Eisenbergiella*	Exclusive breastfeeding	−0.322	191/24	2.4 × 10^−4^	0.018
*Colidextribacter*	Exclusive breastfeeding	−0.398	191/40	3.2 × 10^−4^	0.022
*Akkermansia*	Exclusive breastfeeding	−0.550	191/124	1.3 × 10^−3^	0.066
Uncultured *Lachnospiraceae*	Exclusive breastfeeding	−0.188	191/20	1.4 × 10^−3^	0.069
*Haemophilus*	Exclusive breastfeeding	0.463	191/141	1.7 × 10^−3^	0.073
*Staphylococcus*	Exclusive breastfeeding	0.293	191/56	1.8 × 10^−3^	0.073
*Incertae_Sedis*	Exclusive breastfeeding	−0.358	191/99	1.6 × 10^−3^	0.073
*Flavonifractor*	Partial breastfeeding	−0.909	191/145	7.2 × 10^−7^	1.5 × 10^−4^
*Haemophilus*	Partial breastfeeding	0.740	191/141	1.3 × 10^−5^	0.001
*Lachnoclostridium*	Partial breastfeeding	−0.860	191/146	5.8 × 10^−5^	0.005
*Lactococcus*	Partial breastfeeding	−0.258	191/29	5.6 × 10^−4^	0.031
*Alistipes*	Pre-pregnancy BMI	0.206	191/99	4.0 × 10^−4^	0.025
*Lachnospira*	Age at sample collection	0.171	191/84	5.7 × 10^−4^	0.032

^1^ Model was adjusted for infant antibiotic use, sex, infant birth weight, delivery mode, age at fecal sample collection, infant probiotic supplement and pre-pregnancy BMI. Not breastfeeding is the reference for the breastfeeding status in the regression model. ^2^ q-value is the FDR (Benjamini–Hochberg) adjusted *p*-value. q-value <  0.1 for multiple comparisons was considered statistically significant and included in the table.

**Table 5:** MaAsLin Analysis Results: Associations of infant feeding practices and gut microbiome taxa at genus level. adjusted by covariates within exclusively breastfed and no breastfed infants ^1^.

Breastfeeding Status	Taxonomy at Genus Level	Meta Data Value	Coefficient	N/N Not 0	*p*-Value	q-Value ^2^
Exclusively (N = 88)	*Haemophilus*	Vitamin D supplement (Yes)	−0.683	88/74	6.7 × 10^−5^	0.058
	*Faecalitalea*	Probiotic supplement (Yes)	1.718	60/9	2.9 × 10^−9^	2.4 × 10^−6^
Not breastfeeding (N = 60)	Uncultured *Lachnospiraceae*	Probiotic supplement (Yes)	1.269	60/10	5.1 × 10^−5^	0.021
	*Alistipes*	Pre-pregnancy BMI	0.431	60/27	2.6 × 10^−4^	0.072

^1^ Both Models were adjusted for infant antibiotic use, sex, infant birth weight, delivery mode, age at fecal sample collection, infant probiotic supplement and pre-pregnancy BMI. ^2^ q-value is the FDR (Benjamini–Hochberg) adjusted *p*-value. q-value  <  0.1 for multiple comparisons was considered statistically significant and results were included in the table.

## Data Availability

The data presented in this study are available on request from the corresponding author. The data are not currently publicly available due to ECHO data sharing policy.

## Supplementary Materials

The following supporting information can be downloaded at: https://www.mdpi.com/article/10.3390/nu14010202/s1, Table S1: results of PERMANOVA for all infants on Weighted UniFrac distances, Table S2: results of PERMANOVA for exclusively breastfed infants on Bray–Curtis distances, Table S3: results of PERMANOVA for no breastfed infants on Bray–Curtis distances, Figure S1: infant alpha diversity by different infant feeding practices, and Figure S2: association between breastfeeding status and relative abundance of 8 dominant genera.
