# Supplementary material for: Vitamin D Supplementation in Exclusively Breastfed Infants Is Associated with Alterations in the Fecal Microbiome
Source: Nutrients. 2022 Jan 1;14(1):202. doi: 10.3390/nu14010202 (PMC8747039; doi:10.3390/nu14010202)
Supplement: Supplementary file 1 [file nutrients-14-00202-s001.zip › nutrients-1499177-supplementary.pdf]

SUPPLEMENTARY MATERIAL

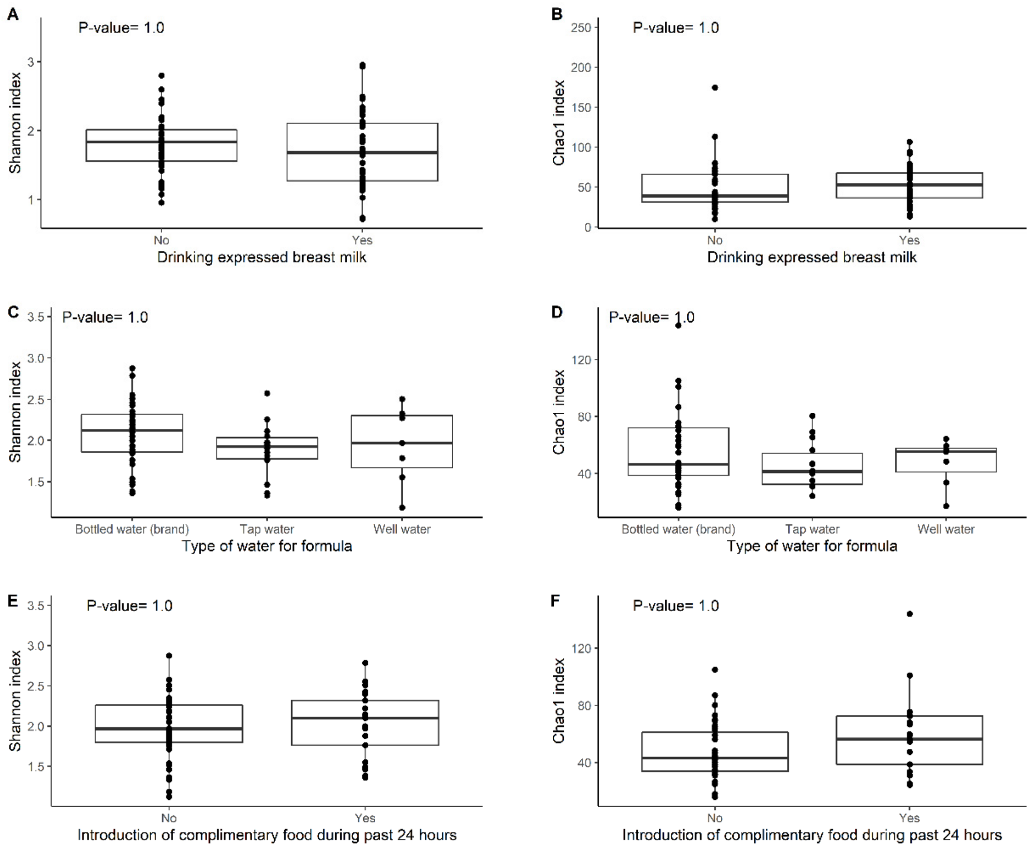

Figure S1. Infant alpha diversity by infant different feeding practices. FDR adjusted p-value for alpha diversity was displayed in upper-left. (A) The Shannon index was used for alpha diversity. Only exclusively breastfed infants were included in this subgroup analysis (N=92). Group differences were tested by Wilcoxon rank test. (B) The Chao1 index was used for alpha diversity. Only exclusively breastfed infants were included in this subgroup analysis (N=92). (C) The Shannon index was used for alpha diversity. Only no breastfed infants were included in this subgroup (N=60). Group differences were tested by Kruskal-Wallis test. We then performed post hoc test for multiple comparisons and no significant associations was found. (D) The Chao1 index was used for alpha diversity. Only no breastfed infants were included in this subgroup (N=60). Group differences were tested by Kruskal-Wallis test. We then performed post hoc test for multiple comparisons and no significant associations was found (N=92). (E) The Shannon index was used for alpha diversity. Only no breastfed infants were included in this subgroup (N=60). Group differences were tested by Kruskal-Wallis test. We then performed post hoc test for multiple comparisons and no significant associations was found. (F) The Chao1 index was used for alpha diversity. Only no breastfed infants were included in this subgroup (N=60). Group differences were tested by Kruskal-Wallis test. We then performed post hoc test for multiple comparisons and no significant associations was found (N=60).

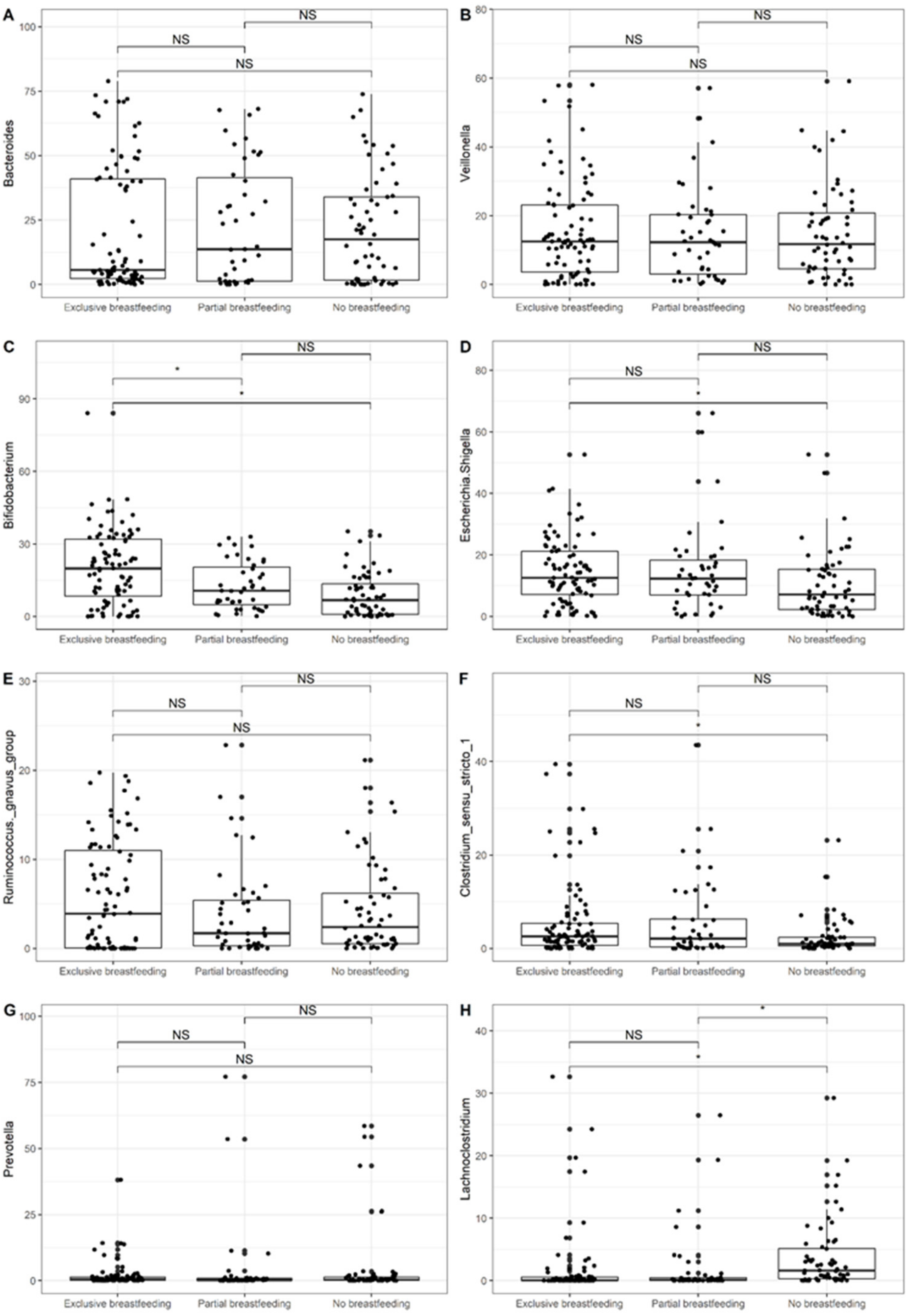

Figure S2. Association between breastfeeding status and 8 most abundant genera. Group differences were tested by Kruskal-Wallis test. Wilcoxon rank test was used for post hoc test. P-value was adjusted by Bonferroni correction.

* indicates the adjusted p-value <0.05

NS indicates the adjusted p-value ≥ 0.05

Table S1. Results of Permutational Multivariate Analysis of Variance (PERMANOVA)[1] with 999 permutations on Weighted UniFrac distances

Variable	F value	R^2	p-value
Breastfeeding during past week	4.8	4.70%	0.001*
Gestational age	4.1	2.10%	0.007*
Infant sex	1.7	0.80%	0.14
Delivery mode (vaginal vs C-section)	6.0	3.00%	0.001*
Infant weight at delivery	0.4	0.20%	0.85
Infant probiotic supplement during past 24 hours	0.7	0.40%	0.57
Infant had any antibiotics since birth	0.9	0.90%	0.50
Maternal educational level	2.3	1.10%	0.06
Maternal pre-pregnancy BMI (continuous)	0.3	0.10%	0.95

* indicates the p-value <0.05

Table S2. Results of Permutational Multivariate Analysis of Variance (PERMANOVA)[1] with 999 permutations for exclusively breastfed infants on Bray-Curtis distances

Variable	F value	R^2	p-value
Gestational age	1.1	1.20%	0.31
Infant sex	0.9	1.00%	0.46
Delivery mode (vaginal vs C-section)	3.0	3.50%	0.01*
Infant weight at delivery	0.3	0.40%	0.92
Infant probiotic supplement during past 24 hours	1.0	1.10%	0.41
Infant had any antibiotics since birth	0.8	0.90%	0.54
Maternal educational level	0.7	0.80%	0.67
Maternal pre-pregnancy BMI (continuous)	0.3	0.30%	0.95
Infant vitamin D supplement in the past 24 hours	2.9	3.40%	0.02*
Feeding with expressed breast milk	0.3	0.30%	0.95

* indicates the p-value <0.05

Table S3. Results of Permutational Multivariate Analysis of Variance (PERMANOVA)[1] with 999 permutations for not breastfed infants on Bray-Curtis distances

Variable	F value	R^2	p-value
Gestational age	1.1	2.00%	0.31
Infant sex	1.2	2.10%	0.29
Delivery mode (vaginal vs C-section)	1.3	2.30%	0.21
Infant weight at delivery	0.4	0.70%	0.94
Infant probiotic supplement during past 24 hours	0.7	1.20%	0.68
Infant had any antibiotics since birth	0.4	0.70%	0.95
Maternal educational level	2.4	4.10%	0.02*
Maternal pre-pregnancy BMI (continuous)	0.6	1.10%	0.75
Water type for formula	1.1	7.40%	0.39
Introduction of complementary food	0.8	1.40%	0.66

* indicates the p-value <0.05
